# Development of an ELISA and Immunochromatographic Strip for Highly Sensitive Detection of Microcystin-LR

**DOI:** 10.3390/s140814672

**Published:** 2014-08-12

**Authors:** Liqiang Liu, Changrui Xing, Huijuan Yan, Hua Kuang, Chuanlai Xu

**Affiliations:** State Key Lab of Food Science and Technology, School of Food Science and Technology, Jiangnan University, Wuxi, JiangSu 214122, China; E-Mails: raxray@gmail.com (L.L.); da_rui12345@163.com (C.X.); yanhuijuan7@163.com (H.Y.); xcl@jiangnan.edu.cn (C.X.)

**Keywords:** microcystin-LR, ELISA, immunochromatographic biosensor

## Abstract

A monoclonal antibody for microcystin–leucine–arginine (MC-LR) was produced by cell fusion. The immunogen was synthesized in two steps. First, ovalbumin/ bovine serum albumin was conjugated with 6-acetylthiohexanoic acid using a carbodiimide EDC (1-ethyl-3-[3-dimethylaminopropyl]carbodiimide hydrochloride)/ NHS (N-hydroxysulfosuccinimide) reaction. After dialysis, the protein was reacted with MC-LR based on a free radical reaction under basic solution conditions. The protein conjugate was used for immunization based on low volume. The antibodies were identified by indirect competitive (ic)ELISA and were subjected to tap water and lake water analysis. The concentration causing 50% inhibition of binding of MC-LR (IC_50_) by the competitive indirect ELISA was 0.27 ng/mL. Cross-reactivity to the MC-RR, MC-YR and MC-WR was good. The tap water and lake water matrices had no effect on the detection limit. The analytical recovery of MC-LR in the water samples in the icELISA was 94%–110%. Based on this antibody, an immunochromatographic biosensor was developed with a cut-off value of 1 ng/mL, which could satisfy the requirement of the World Health Organization for MC-LR detection in drinking water. This biosensor could be therefore be used as a fast screening tool in the field detection of MC-LR.

## 1. Introduction

Microcystins (MCs) are a series of structurally related hepta- and pentacyclic peptide toxins with molecular weights between 900 and 1200 produced by certain freshwater cyanobacteria, including *Oscillatoria agardhii*, *Nodularia spumigena*, *Microcystis aeruginosa* and *Anabaena flos-aquae* [1]. Contamination with cyanobacteria has a severe effect on drinking water safety and public health.

As tumor promoters, MCs cause a range of adverse effects in animals and humans [2]. The MCs are concentrated in liver cells through a bile-acid-type carrier mechanism. By specifically binding to protein phosphatases 1 and 2A in the liver, MCs inhibit the function of these two key enzymes in cellular regulation. Long-term accumulation in the human body causes a high incidence of primary liver cancer [3].

Some organisms degrade MCs and nodularin in water bodies. They are also subject to acid hydrolysis, oxidation, reduction and ozonization [4]. However, the MCs in solution are relatively stable to heat and chemical breakdown by hydrolysis, even under high-pH conditions [5], so conventional water treatment methods cannot destroy or remove the MCs [6]. Some outbreaks of poisoning by MCs have occurred in chlorinated tap water supplies, indicating that monitoring of tap water is needed [3]. The World Health Organization (WHO) recommended a limit of 1 ng/mL for cyanobacterial toxins in drinking water for long-term exposure [7]. In order to monitor environmental contamination with MCs and reduce exposure to humans and animals, highly sensitive and specific methods have been developed for detection of MCs [8,9].

The most common analytical method for the detection of MCs is high-performance liquid chromatography (HPLC) [10,11]. This approach has been extensively applied to detection of toxins in cyanobacterial material. HPLC is sensitive and used for quantitative analyses, but requires expensive instruments and skilled operators. Capillary electrophoresis and related techniques have also been considered for quantification of MCs. However, these methods lack sensitivity compared with HPLC and are not suitable for routine and on-site monitoring of water samples. The extent of the inhibition of protein phosphatase-1 and 2A by the MCs has been used as a measure of toxin concentrations in environmental samples [12]. The assay needs ^32^P-radiolabeled substrates and is not suitable for most routine laboratories, and some reagents are expensive. Recently, nanotechnology combined with antibody–antigen reaction has shown advantages with regard to detection limits [13–23]. However, these methods are still unsuitable for field detection and some processes are complicated and difficult for untrained personnel to operate.

ELISAs have been developed as simple and fast methods for semiquantitative determination of MCs [1,24–38]. Antibodies were produced [28] and used for determining MC-LR in environmental samples. Although only a limited number of MCs were tested for their cross-reactivity, the ELISA was useful as a screening tool. Zeck *et al.* have developed a generic MC immunoassay based on monoclonal antibodies (mAbs) against ADDA [39], and a highly sensitive immunoassay based on an mAb specific for [4-arginine]MCs [40]. In a direct competitive ELISA based on MC10E7 (the name of the cell line), 50% inhibitory concentration (IC_50_) for MC-LR was 0.06 ng/mL [40]. The class-specific antibodies AD4G2 (the name of cell line) could recognize ADDA and ADDA derivatives with good sensitivity [39].

Here, we synthesized the immunogen with reference to the method of Zeck *et al.* [40] with some modifications. In monitoring the immune response and screening cell culture supernatants, we developed a highly sensitive indirect competitive (ic)ELISA against MC-LR. Based on the mAb screened, a highly sensitive ELISA and an immunochromatographic assay for the semi-quantification of MCs were established [41–47]. The biosensor systems were fast and reproducible for direct detection of low-level MCs in real samples, without an extraction step. The detection time was within 10 min and could be used for field detection of MCs.

## 2. Experimental Section

### 2.1. Materials and Reagents

MC-LR [CAS registry number 101043-37-2; molecular weight (MW) 995.17 g/mol], MC-RR (CAS registry number 111755-37-4; MW 1038.2 g/mol), and other MC analogs were purchased from Express Technology Co. Ltd. (Beijing, China) MC standards were prepared as the concentration of 1 mg/mL in anhydrous N,N-dimethylformamide (DMF) and were kept at 4 °C in amber glass vials. 6-Acetylthiohexanoic acid (CAS registry number 80441-57-2; MW 190.26 g/mol), ovalbumin (OVA), bovine serum albumin (BSA), iodoacetate and Ellman's reagent were purchased from Sigma (St. Louis, MO 63103, USA). Horseradish peroxidase (HRP)-conjugated AffiniPure Goat Anti-Mouse IgG (H+L) was purchased from Jackson ImmunoResearch Laboratories (Beijing, China).

### 2.2. Buffers and Solutions

Carbonate buffer solution (CBS), 50 mM sodium carbonate–bicarbonate buffer, pH 9.6; phosphate buffered saline (PBS), 10 mM sodium phosphate buffer, pH 7.4, with 140 mM NaCl; phosphate buffered saline tween-20 (PBST), PBS containing 0.05% (v/v) Tween 20; and PBST-Gelatin, PBST containing 0.1% (w/v) gelatin.

### 2.3. Synthesis of the MC-LR Immunogen

**Scheme 1.**
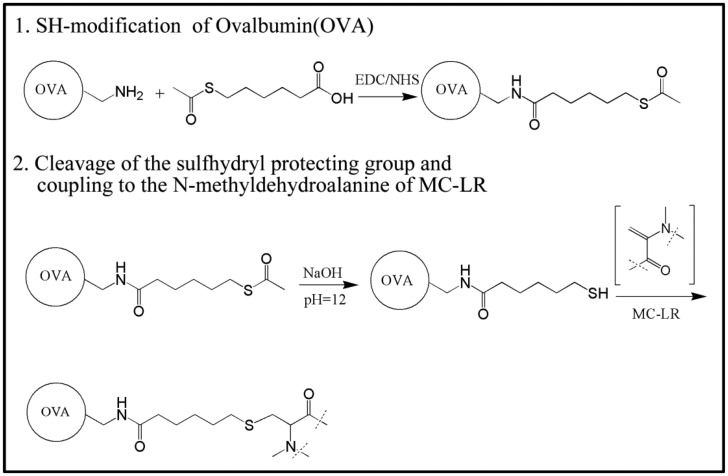
Synthesis of MC-LR–OVA immunogen.

MC-LR was synthesized according to Zeck *et al.* [39], with some modifications. OVA was used as the carrier protein and conjugated with 6-acetylthiohexanoic acid with the EDC/NHS method. The conjugate was purified by dialysis and conjugated with MC-LR at pH 12. As shown in Scheme 1, OVA was first SH-modified by the conjugate with 6-acetylthiohexanoic acid. Fifty microliters of 6-acetylthiohexanoic acid was added to the mixture of 0.1 M 2-(N-morpholino)ethanesulfonic acid buffer (MES buffer, 2 mL) and DMF (3 mL). Then, EDC (10 mg) and NHS (10 mg) were added and activated for 6 h. The activated solution was added to CBS (10 mL) containing OVA (20 mg). The reaction was allowed to proceed overnight. The SH-modified protein was dialyzed for 3 days with PBS. The protein was identified by polyacrylamide gel electrophoresis and detected by monitoring the SH groups with Ellman's reagent. The pH of the thiolated protein in PBS was adjusted to 12 with 5 mol/L NaOH. We immediately added MC-LR (1 mg) dissolved in acetonitrile (1 mL) containing 1% dimethylsulfoxide (DMSO). The reaction mixture was stirred overnight at room temperature. Three milligrams of iodoacetate was added to the reaction mixture for 15 min. MC-LR–OVA were dialyzed for 3 days with PBS. MC-LR–BSA was synthesized by the same process and used as the coating reagent in the ELISA and immunochromatographic assay.

### 2.4. Immunization and Hybridoma Screening

BALB/c mice were used for the animal experiments. MC-LR–OVA was emulsified with Freund's complete adjuvant (for the first time) and Freund's incomplete adjuvant (booster immunization) at intervals of 1 month. Blood was collected 10 days later after the third immunization to monitor the titer and affinity of MC-LR-specific antibodies. After the fifth immunization, one of the mice shown high affinity to MC-LR measured by icELISA. This mouse was boosted with 20 μg immunogen and killed for cell fusion 3 days later. The spleen was removed in a sterile environment and ground through a cell sieve. The spleen cells were washed three times and fused with the pre-collected SP20 cells using PEG 1500. The ratio of spleen cells to SP20 cells was 10:1. After cell fusion, the hybridoma was cultured with hypoxanthine aminopterin thymidine (HAT) medium plated on 96-well plates.

### 2.5. Screening of Cell Culture Supernatants

All the cell culture fluid in the wells was added to the plates precoated with MC-LR–BSA for positive hybridoma cell screening. The positive cells combined with MC-LR–BSA and were tested for their affinity with free MC-LR by icELISA. Microtiter plates were coated with MC-LR–BSA overnight at 4 °C with 100 μL/well at a concentration of 0.1 μg/mL in CBS. After washing four times with PBST, the plates were blocked with blocking buffer in CBS at 37 °C for 2 h with 200 μL/well. The plates were washed and air dried. The culture fluid in the wells was tested for reaction with MC-LR. Plates were incubated with different concentrations of MC-LR (diluted in 0.01 M PBS) and blank buffer (0.01 M PBS), together with cell culture supernatant diluted 1:4 in PBST-Gelatin, at 37 °C for 30 min. Serum from the mice for fusion (1:1000) was also added as a positive control. The plates were washed four times with PBST and incubated with HRP AffiniPure Goat Anti-Mouse IgG (1:3000) for 30 min. After the plate was washed four times, 3,3′,5,5′-tetramethylbenzidine (TMB) substrate solution was added. After incubation at 37 °C in the oven for 15 min, the reaction was stopped by 0.1 mL 2 N H_2_SO_4_. The absorbance at 450 nm was determined by a microtiter plate reader.

### 2.6. Production and Purification of mAbs

The clones shown good sensitivity for MC-LR and were subcloned three times by successive limiting dilutions, and the hybridoma cells were cultured on a large scale and cryopreserved. The hybridoma cells (1 × 10^7^) were injected into the peritoneal cavity of mice for large-scale production of mAbs. We purified the mAbs from ascites fluid as follows: centrifugation, membrane filtration, saturated ammonium sulfate precipitation, and protein-G column chromatography. The mAbs were eluted with 100 mM glycine–HCl (pH 2.5) from the column and neutralized by 0.1 volume of 1 M Tris (pH 8.0).

### 2.7. ELISA

The protocol for the icELISA was the same as that described previously for screening of cell culture supernatants, except that the antibody was diluted in 0.01 M PBST-gelatin (0.1 μg/mL), and a series of MC-LR standard solutions (0, 0.02, 0.05, 0.1, 0.2 0.5, 1 and 2 ng/mL) was tested. Each calibration point of the standard curves and each sample value was determined by the mean of the data (*n* = 3). Standard curves were obtained by a four-parameter fitting function.

### 2.8. Gold Nanoparticle (GNP)-Labeling of mAbs

Before GNP labeling of mAbs, 0.1 M K_2_CO_3_ was used to adjust the pH of the 20 nm GNP synthesized in our laboratory. For 10 mL GNP solution in a 50-mL centrifuge tube, 40 μL K_2_CO_3_ was used. mAb (0.1 mg) in pH 8.0 borate buffer solution (0.5 mL) was added dropwise, and the conjugation process was maintained at room temperature for 1 h. BSA (50 mg) was dissolved in 1 mL ultrapure water and added slowly to the GNP solution for blocking and stabilizing the GNP labeling of mAb. After 2 h, the GNP–mAb was centrifuged at 8000 rpm for 25 min. After decanting the supernatant, the precipitate was dissolved in 5 mL PBS containing 0.2% BSA.

### 2.9. Fabrication of Immunochromatographic Strip

The immunochromatographic strip consisted of a sample pad, nitrocellulose membrane, polystyrene backing card, and absorption pad. They were assembled layer by layer. The test and control zones were dispensed by the BJQ3000 Membrane dispenser machine (XinqidianGene-technology Co. Ltd., Beijing, China). MC-LR–BSA (2 mg/mL) was used in the test zone to detect MCs in samples and Goat Anti-Mouse IgG (0.5 mg/mL) was used in the control zone. The width between the test and control zones on the nitrocellulose was 0.5 mm and the speed was 1 μL/cm. The nitrocellulose membrane was air dried before use. The sample pad was immersed in PBS containing 1% BSA and 0.2% Tween 20, and air dried at 37 °C for 4 h. The assembled strip was placed into a plastic cartridge before use.

### 2.10. Immunochromatographic Assay

The biosensor systems to quantify MCs were based on the competitive/inhibitory interaction between free MC-LR in samples and the MC-LR–BSA for GNP-labeled mAb. The intensity of the test zone reflected the amount of uncombined GNP–mAb with free MC-LR. The more MC-LR in the sample, the more GNP–mAb interacted and the less color intensity on the test zone accumulated by MC-LR–BSA. The color intensity of the test zone was inversely proportional to the concentration of MC-LR in the samples. A series of MC-LR standard solutions (0, 0.2, 0.5 and 1 ng/mL) was added to the GNP–mAb and allowed to react for 5 min at room temperature. The reaction solution was added to the strip. The detection results were judged by naked eye 5 min later.

### 2.11. Sample Analysis

Different water samples were spiked with different amounts of MC-LR for sample analysis. At first, the original concentration of MC-LR in different water samples was measured by HPLC, including drinking barreled water from the supermarket, tap water from our laboratory, and lake water from Wuxi Taihu Lake, China. Lake water samples were analyzed with sample pretreatment. Lake water samples were filtered through a 0.45-μm nitrocellulose membrane immediately after sampling. Different concentrations of MC-LR included in the measuring range were spiked and detected by ELISA and the strip biosensor.

## 3. Results and Discussion

### 3.1. MC-LR Conjugate Preparation, Immunization, and Serum Detection

MC-LR–OVA was used as an immunizing conjugate at a dose of 20 μg per immunization. From the third immunization, serum was collected from the mice. Antisera were separated by centrifugation from whole blood. The data tested by icELISA (MC-LR–BSA was diluted to 0.1 μg/mL in CBS as coating reagent) with serum dilutions of 1:1000, 1:2000 and 1:4000 shown that mouse 25 had a high affinity with free MC-LR. Serum from the fifth immunization shown an IC_50_ of ∼5 ng/mL for MC-LR. The signal was decreased from 1.5 abs to 0.7 abs when 5 ng/mL MC-LR standards in PBS were tested. Because mouse 25 shown a high concentration of antibody specific for MC-LR, it was used for cell fusion.

### 3.2. Isolation of Hybridoma Cell Line Producing Antibodies to MC-LR

Hybridized cells were cultured on 12 96-well plates. All wells had growing cells and 56 of 1152 wells were positive when tested by indirect ELISA. To detect their reactivity to free MC-LR, the mAbs in the cell culture supernatant were detected by icELISA. MC-LR standards (1 ng/mL) in PBS were used to screen the sensitivity of positive hybridized cells. Culture supernatant from 10 wells shown high-sensitivity antibodies that could react with free MC-LR. The hybridized cells in these wells were recloned twice. Finally, one cell line (MC4G7) was selected to enlarge the culture and obtain larger amounts of mAb.

### 3.3. Evaluation of Standard Curve and Affinity Constant Measurement

**Figure 1.**
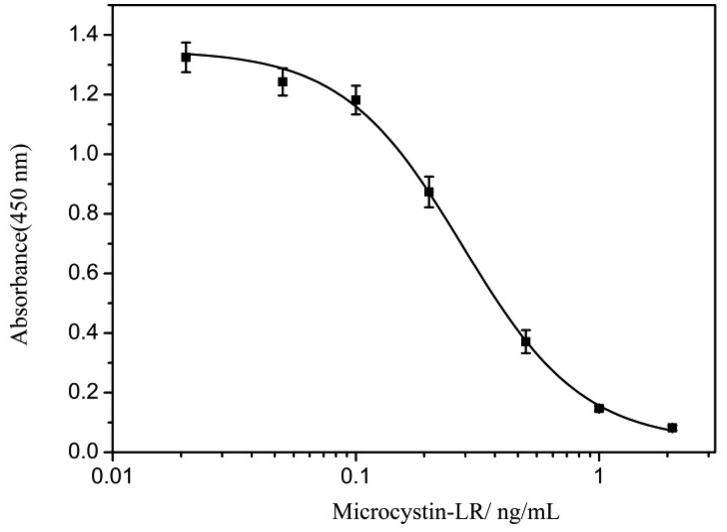
Standard curve for measurement of MC-LR.

The standard curve for measurement of MC-LR based on the mAb MC4G7 is shown in Figure 1. The absorbance is plotted against the log of the MC-LR concentration from 0.02 to 2 ng/mL. The IC_50_ was 0.27 ng/mL and the detection range was 0.11–0.7 ng/mL (IC_20_ to IC_80_). This antibody was suitable for measurement of MC-LR in water samples as the provisional guideline value of 1 ng/mL MC-LR proposed by the WHO. The affinity constant was determined as 1.124 × 10^9^.

### 3.4. Cross-reactivity

The MC variants including MC-RR, MC-YR, MC-LW, MC-LF, MC-LA, MC-LY, MC-WR, MC-HtyR and [D-Asp^3^] MC-RR were examined for cross-reactivity with MC-LR (Table 1). As shown by the IC_50_ and cross-reactivity, the MC variants containing [4-arginine] were recognized by the antibody MC4G7. This indicated that the amino acid residues at position 4 of the MCs were an important antigenic site for antibody binding.

### 3.5. Sample Analysis by ELISA

To evaluate the applicability of the developed immunoassay, drinking, tap and lake water samples were spiked with different amounts of MC-LR. The lake water was filtered through a 0.45-μm membrane and detected directly. The samples were measured unspiked by HPLC, which shown that they were not contaminated (Table 2). Recovery of the spiked samples was 94%–110%. The repeatability was evaluated by the detection of different concentrations of MC-LR (Table 3). The intra- and inter-assay coefficients of variation were 2.1%–3.9% and 4.3%–5.2%, respectively. The variation was low and acceptable and shown that this assay was repeatable. The developed ELISA had high sensitivity and accuracy for detection of MCs in environmental water samples.

### 3.6. Fabrication and Characterization of Immunochromatographic Strip

**Figure 2.**
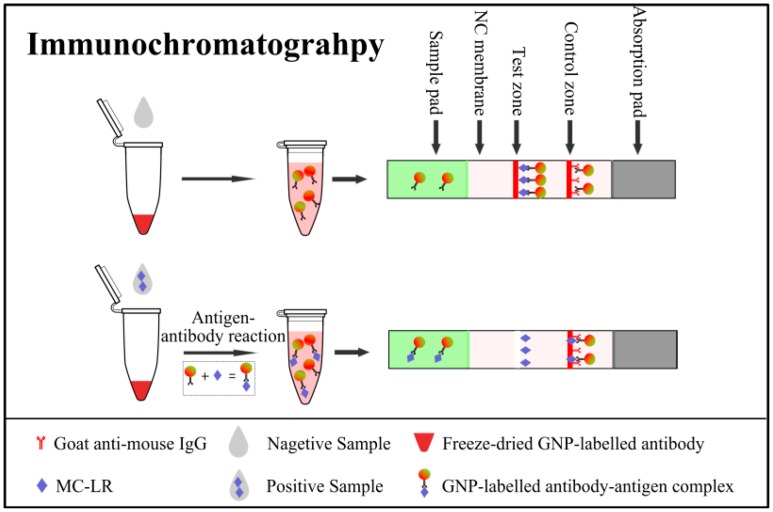
Schematic diagram of the immunochromatography for MC-LR detection.

For fast and on-site detection, we chose GNP as the detection signal in lateral-flow-type immunochromatography. Signal generation in the immunochromatography assay based on GNP-labeled antibody did not require a substrate for color development. The results could be judged by the naked eye, taking advantage of the red color of GNP. Immunochromatography with GNP-labeled antibody was extensively developed and could be used for semi-quantification of MCs. Figure 2 shows a schematic diagram of the immunochromatography assay.

The sample pad was pretreated by PBS buffer containing 1% BSA and 0.2% Tween 20 in order to prevent nonspecific binding and matrix interference. The GNP-labeled antibody was freeze-dried in the centrifuge tube. The detection process was as follows. One hundred microliters of the sample solution was added to the centrifuge tube containing GNP-labeled mAb and vortexed for 5 s. Then, the sample and GNP-labeled mAb mixture were allowed 5 min reaction at room temperature. The MC-LR bound to the GNP-labeled mAb and formed an antigen–antibody complex before being applied to the sample pad. The amount of this complex increased with the amount of MC-LR in the sample solution. For the immunochromatography assay, 80 μL sample mixture was loaded onto the sample pad of the assembled strip. The sample solution was running on the nitrocellulose membrane and the test and control zones appeared as thin red lines. If large amounts of MC-LR were present in the sample solution, much of the GNP-labeled mAb bound to MC-LR to form the antigen–antibody complex, leaving only a small amount of unreacted GNP-labeled mAb captured by MC-LR–BSA in the test zone. The color intensity in the test zone was light red in color. If the sample contained no MC-LR, the GNP-labeled mAb was captured in the test zone, resulting in a deep red color. After 5 min, the results were judged by the naked eye.

**Figure 3.**
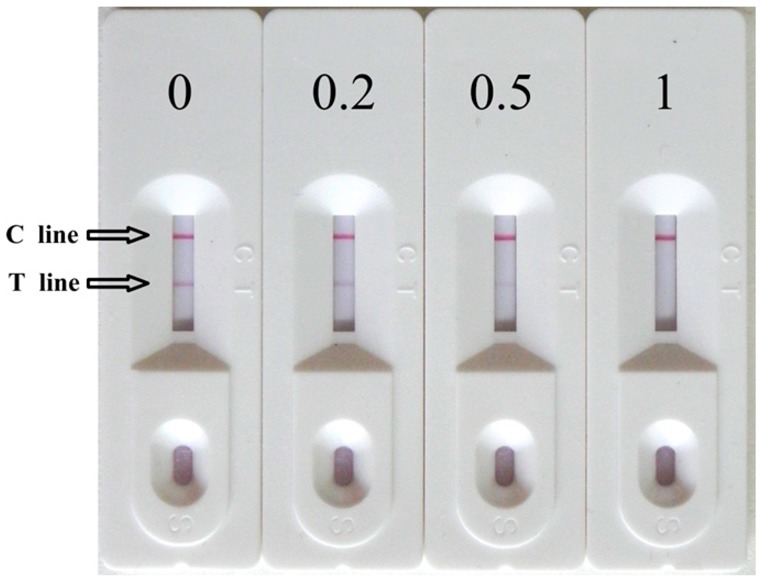
Typical photo image of detection MC-LR by strip sensor in PBS.

A series of MC-LR standard solutions (0, 0.2, 0.5, 1 ng/mL in PBS) was measured (Figure 3). When the concentration of MC-LR in the sample solution was 1 ng/mL, no test line was observed. The cut-off value (a threshold MC-LR concentration at which the test line disappeared) of the immunochromatography assay was 1 ng/mL.

### 3.7. Sample Matrix Effects on Immunochromatographic Strip

**Figure 4.**
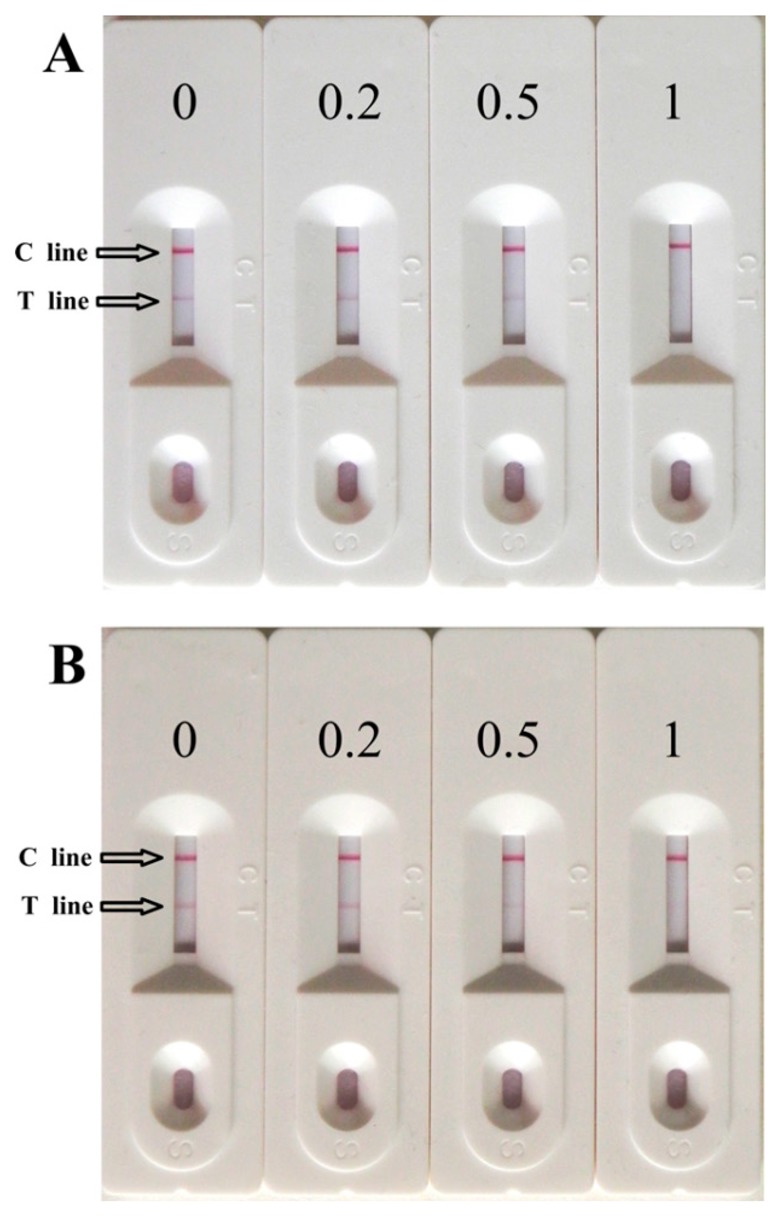
Typical photo image of detection MC-LR by strip sensor in tap water (**A**) and lake water (**B**).

In order to verify if this immunochromatography assay could be used for detection of MC-LR in environmental water samples, tap and lake water spiked with different concentrations of MC-LR. The results were consistent with those using PBS (Figure 4). Although the strip did not work well below 0.2 ng/mL (the color intensity change in the test line could not be identified by the naked eye; data not shown), the strip sensor worked well and no test line could be seen when 1 ng/mL MC-LR was spiked. We conducted reproducibility tests at 1 ng/mL of free MC-LR in different water samples with 90 independent experiments (Table 4). The reproducibility in the immunochromatographic strip was good and this method could meet the requirement of the WHO for MCs in drinking water. The strip biosensor is a useful tool for on-site detection of MCs in drinking or environmental water.

## 4. Conclusions

In our study, a simple method for the synthesis of the conjugate MC-LR–OVA/BSA was developed and used to produce anti-MC-LR mAbs. The four MC-LR analogs showed high cross-reactivity with the MC-LR mAbs, which was consistent with previous results. The icELISA method was used to detect MC-LR in tap and lake water. The recovery was good and the detection limit satisfied the requirement of the WHO. In addition, we successfully developed an immunochromatographic biosensor with fast and convenient characterization for semi-quantification of MCs. This biosensor showed good sensitivity, with a cut-off value of 1 ng/mL, allowing for primary screening of MCs in environmental water in the field by nonprofessional personnel.

## Figures and Tables

**Table 1.** IC_50_ and cross-reactivities.

**Analyte**	**IC_50_ (ng/mL)**	**Cross-reactivities (%)**
MC-LR(Microcystin-Leucine-Arginine)	0.27	100%
[D-Asp^3^] MC-RR([D-Asp^3^] Microcystin-Arginine-Arginine)	0.18	150%
MC-YR(Microcystin-Tyrosine-Arginine)	0.22	123%
MC-RR(Microcystin-Arginine-Arginine)	0.28	96%
MC-WR(Microcystin-Tryptophan-Arginine)	0.29	93%
MC-HtyR(Microcystin-Homotyrosine-Arginine)	0.6	45%
MC-LY(Microcystin-Leucine-Tyrosine)	>300	<0.1%
MC-LW(Microcystin-Leucine-Tryptophan)	>300	<0.1%
MC-LF(Microcystin-Leucine-Phenylalanine)	>300	<0.1%
MC-LA(Microcystin-Leucine-Alanine)	>300	<0.1%

**Table 2.** MC-LR recovery values from spiked water sample by ELISA.

**Water Sample**	**MC-LR Added to the Sample (ng/mL)**	**MC-LR Determined by ELISA (ng/mL)**	**Recovery of MC-LR (%)**
Drinking water	0.2	0.207	103
	0.5	0.523	105
Tap water	0.2	0.187	94
	0.5	0.532	107
Lake water	0.2	0.212	106
	0.5	0.553	110

**Table 3.** Intra- and inter-assay variation for MC-LR detection.

**Concentration (ng/mL)**	**Intra-assay ^a^**		**Inter-assay ^b^**	
	
	***n***	**CV *^c^* (%)**	***n***	**CV ^c^ (%)**
0.2	3	3.9	5	5.2
0.5	3	2.1	5	4.3

a Intra-assay variation was calculated from 3 replicates on a single day.

b Inter-assay variation was calculated from triplicates on 5 different days.

c CV: coefficient of variation.

**Table 4.** Performance Strip Test.

**Spiked Concentrations (ng/mL)**	**Water Samples**		

	**PBS Solution *n* = 30**	**Tap Water *n* = 30**	**Lake Water *n* = 30**
0	+ *^a^*	+	+
1	− *^b^*	−	−

a Negative result. The test line is obviously observed.

b Positive result. No test line is observed.
